# Infection incidence, timing, and predictors in newly diagnosed multiple myeloma: a real-world retrospective cohort study

**DOI:** 10.3389/fmed.2026.1829429

**Published:** 2026-05-14

**Authors:** Ozlem Candan, Narmin Naghizada, Tekin Tuncel, Beyza Melek Palaz, Mustafa Alperen Tunc, Derya Demirtas, Ahmet Mert Yanik, Arda Bayar, Secil Salim, Fatma Temiz, Ceren Uzunoglu Guren, Fatma Arıkan, Meral Ulukoylu Menguc, Asu Fergun Yilmaz, Isik Atagunduz, Zekaver Odabası, Tayfur Toptas, Ayse Tulin Tuglular

**Affiliations:** 1 Division of Hematology, Department of Internal Medicine, Istanbul Kanuni Sultan Suleyman Training and Research Hospital, Istanbul, Türkiye; 2 Department of Internal Medicine, Pendik Training and Research Hospital, Marmara University School of Medicine, Istanbul, Türkiye; 3 Department of Infectious Diseases and Clinical Microbiology, Pendik Training and Research Hospital, Marmara University School of Medicine, Istanbul, Türkiye; 4 Division of Oncology, Department of Internal Medicine, Pendik Training and Research Hospital, Marmara University School of Medicine, Istanbul, Türkiye; 5 Division of Hematology, Department of Internal Medicine, Adana City Training and Research Hospital, Adana, Türkiye; 6 Division of Hematology, Department of Internal Medicine, Bursa City Training and Research Hospital, Bursa, Türkiye; 7 Division of Hematology, Department of Internal Medicine, Pendik Training and Research Hospital, Marmara University School of Medicine, Istanbul, Türkiye

**Keywords:** comorbidities, induction therapy, infections, multiple myeloma, real-world study, risk stratification

## Abstract

**Background:**

Infections are a leading cause of morbidity and mortality in multiple myeloma (MM), particularly during the induction phase. Identifying real-world infection patterns and predictors is crucial for guiding preventive strategies.

**Methods:**

This retrospective cohort study included 161 newly diagnosed MM patients who received standard induction therapy at a single tertiary center between 2016 and 2024. Clinical, laboratory, and infection-related parameters were analyzed, with infection during the first 6 months defined as the primary outcome and 12-months analyses as secondary outcomes.

**Results:**

Infections occurred in 50.9% of patients, with the highest incidence within the first 3 months (27.3%, *p* = 0.004). Pneumonia was the most common type (34.1%), and gram-negative bacteria, particularly *Escherichia coli*, *Klebsiella* spp., and *Pseudomonas aeruginosa*, were predominant. Antibiotic resistance to TMP-SMX and levofloxacin was observed in *E. coli* isolates. Multivariate analysis identified advanced ISS stage (OR: 3.83), diabetes mellitus (OR: 3.64), and chronic kidney disease (OR: 6.01) as independent predictors of infection (*p* < 0.05). Lymphocyte counts were significantly lower in febrile episodes (*p* = 0.040), suggesting a potential association with immune status, although this finding should be interpreted cautiously. Hospitalization was more common in patients with advanced ISS and kidney disease. No significant differences were found between induction regimens.

**Conclusion:**

Infections pose a significant burden during the first year of MM treatment, particularly in high-risk patients during early induction. Readily available clinical parameters can aid in early risk stratification. These findings support a risk-adapted approach to infection prevention based on clinical risk factors, particularly during the early phase of treatment.

## Introduction

Multiple myeloma (MM) is a hematologic malignancy characterized by clonal plasma cell proliferation and profound immune dysfunction. Infections remain a major cause of morbidity and mortality in MM, attributed both to disease-related immune suppression and to treatment-related toxicity (1, 2). Previous studies have reported a 7-fold increase in infection risk among MM patients compared to the general population, particularly during the first 6 months of induction therapy (3, 4).

The pathophysiology of MM includes several factors that predispose patients to infections. Hypogammaglobulinemia, a hallmark of MM, compromises the immune system’s ability to respond against bacterial pathogens effectively (5). Additionally, myelosuppression-induced neutropenia significantly increases susceptibility to bacterial and fungal infections (6). Furthermore, T-cell dysfunction and a reduction in cytotoxic T-cell numbers impair antiviral immunity, leading to an increased incidence of viral infections (7). Additionally, older age and comorbidities such as chronic kidney disease and diabetes compound this risk (8).

While prophylactic antibiotic use has been considered as a strategy to reduce infection-related morbidity in MM, its routine implementation remains controversial. Concerns include toxic side effects, the emergence of resistant microorganisms, and the potential for altered therapeutic response to immunomodulatory agents. These limitations highlight the importance of improved risk stratification to guide more targeted infection prevention strategies (5, 9).

Induction regimens play a crucial role in modulating the risk of infection. Proteasome inhibitors (e.g., bortezomib), immunomodulatory drugs (e.g., lenalidomide), corticosteroids, and monoclonal antibodies (e.g., daratumumab) all contribute to varying degrees of immunosuppression. These therapeutic regimens not only disrupt immune homeostasis but also determine the severity and type of infections observed during treatment (8, 10). In addition to these factors, prolonged hospitalizations and the use of central venous catheters contribute to nosocomial infections, further increasing the infection burden (11).

Common pathogens isolated in MM patients include *Escherichia coli* and *Klebsiella pneumoniae* among gram-negative bacteria, *Staphylococcus aureus* and *Streptococcus pneumoniae* among gram-positive bacteria. Viral infections, particularly herpes zoster and cytomegalovirus (CMV) reactivation, are frequently reported, while fungal infections, such as those caused by *Candida* and *Aspergillus* species, are more commonly observed in advanced disease stages or patients receiving high-dose corticosteroids (3, 12).

The multifactorial nature of infection risk in multiple myeloma, along with the increasing availability of real-world data, makes it crucial to understand the timing and predictors of infectious complications for optimal patient management. In this study, we aimed to evaluate the incidence and temporal distribution of infections during the first 12 months of induction therapy, with a particular focus on the initial 6-months period. By analyzing routinely collected clinical and laboratory parameters, we sought to identify key factors associated with increased infection risk. Our goal was to enable earlier identification of high-risk patients, thereby supporting a more individualized clinical approach. Ultimately, we aimed to generate clinically meaningful insights that could strengthen infection prevention strategies and improve outcomes during this vulnerable phase of treatment.

## Materials and methods

### Study design and patient selection

This retrospective cohort study was conducted at a single tertiary care center and included patients diagnosed with multiple myeloma between January 2016 and December 2024. Eligible patients were 18 years or older, had a new diagnosis of MM, and received standard induction regimens such as bortezomib-based combinations (e.g., VRD, VCD, VD) or lenalidomide-based doublets (e.g., RD). Information regarding daratumumab-containing regimens was collected; however, due to the limited number of patients receiving such treatments, subgroup analysis was not performed. Inclusion required complete clinical and laboratory data at diagnosis and a minimum of 6 months of follow-up. Patients were excluded if they had previously received systemic therapy for MM, had active infections at baseline, received any form of prophylactic antimicrobial treatment, had a confirmed COVID-19 infection, or had missing key data related to infection status or laboratory parameters. These exclusion criteria aimed to reduce confounding from viral epidemics and antimicrobial interventions, resulting in a COVID-negative, non-prophylaxed study population.

### Definition of infections

Infectious episodes were defined as clinically or microbiologically documented events requiring antimicrobial therapy and/or hospitalization. Clinical diagnoses were based on physician assessment of infection-related signs and symptoms, supported by imaging or laboratory findings, in accordance with general diagnostic principles recommended by the Infectious Diseases Society of America (IDSA) (13). In cases without a clearly identifiable focus, infections were classified based on clinical judgment supported by laboratory and radiological findings, and were included if antimicrobial treatment was initiated.

### Data collection

Baseline demographic and clinical data were extracted from the hospital’s electronic medical records. Collected variables included age, sex, and comorbidities such as hypertension, diabetes mellitus, chronic kidney disease, congestive heart failure, coronary artery disease, chronic pulmonary disease [e.g., chronic obstructive pulmonary disease (COPD) or asthma], autoimmune disorders, and a history of prior malignancy.

Peripheral blood neutrophil and lymphocyte counts at the time of infection were also recorded to assess hematologic parameters associated with infectious episodes. These values were obtained from the nearest complete blood count (CBC) performed within 48 h of the documented infection event. This approach reflects the patient’s immune status during infection and allows for the identification of potential hematologic predictors of infection risk.

Disease-related variables, including the International Staging System (ISS) score and the specific induction regimen, were also recorded. ISS was analyzed as an ordinal variable in the statistical analysis. Cytogenetic risk status and lactate dehydrogenase (LDH) levels were excluded from the final analysis due to high rates of missing data; therefore, the R-ISS score could not be reliably calculated, and only the standard ISS system was used. In addition, CMV infections were not systematically assessed, as routine CMV DNA testing was not performed in all patients.

### Outcome measures

The primary outcome was defined as the occurrence of any infectious episode during the first 6 months of induction therapy, as this period represents the highest clinical vulnerability. Secondary outcomes included the incidence and temporal distribution of infections over the full 12-months period, allowing for evaluation of infection patterns throughout the first year of treatment.

Infections were considered present if they were clinically or microbiologically documented and required treatment with antibiotics, antivirals, or antifungals, and/or resulted in hospitalization.

To assess infection patterns over time, all infectious episodes were categorized into three intervals: 0–3 months (early phase), 3–6 months (intermediate phase), and 6–12 months (late phase). This temporal stratification allowed for a more detailed evaluation of infection dynamics throughout the first year of therapy.

### Statistical analysis

Descriptive statistics were used to summarize baseline characteristics. Continuous variables were expressed as medians with interquartile ranges (IQR), while categorical variables were reported as frequencies and percentages. Associations between baseline clinical characteristics and infection status were analyzed using the chi-square or Fisher’s exact test for categorical variables, and the Mann–Whitney U test for continuous variables.

To identify independent predictors of infection, univariate logistic regression analyses were first performed. Variables with *p* < 0.10 in univariate analysis, as well as those considered clinically relevant based on prior literature and clinical judgment, were considered for inclusion in the multivariable logistic regression model. Model selection was based on a combination of statistical significance and clinical relevance.

Logistic regression was selected as the primary analytical method due to the binary nature of the outcome variable (occurrence of infection). The extent of missing data was assessed and transparently reported. Missing data were handled by excluding variables with a high proportion of missing values from the final model, and multicollinearity was assessed prior to model construction.

Due to the retrospective design, time-to-event analysis and recurrent event modeling were not performed; this limitation has been acknowledged.

All statistical analyses were performed using Python (version 3.11.0), with the pandas, scipy, and statsmodels libraries.

### Ethical approval

Ethical approval for this study was obtained from the Clinical Research Ethics Committee of Marmara University School of Medicine (Protocol No: 09.2023.1454).

## Results

### Patient characteristics

A total of 161 patients with newly diagnosed multiple myeloma were included in the study. The median age was 64 years (interquartile range [IQR], 59–73), and 59.0% were male. The most common comorbidities were hypertension (26.7%), diabetes mellitus (23.6%), and chronic kidney disease (15.5%). According to the ISS, 40.4% were classified as stage III, 20.5% as stage I, and 18.6% as stage II. The most frequently administered induction regimen was VCD (52.8%), followed by VRD (23.6%), VD/RD (7.5%), and other combinations (16.1%) (Table 1). A total of 33 patients were documented to have undergone autologous stem cell transplantation at our center during the study period.

**TABLE 1:** Baseline characteristics of patients (*n* = 161).

Characteristic	Median (IQR)/*n* (%)
Age, median (IQR)	64 (59–73)
Sex (Male)	95 (59.0%)
Hypertension	43 (26.7%)
Diabetes mellitus	38 (23.6%)
Chronic kidney disease	25 (15.5%)
ISS stage	
Stage 1	33 (20.5%)
Stage 2	30 (18.6%)
Stage 3	65 (40.4%)
Not available	33 (20.5%)
Induction regimen	
VCD	85 (52.8%)
VRD	38 (23.6%)
VD/RD	12 (7.5%)
Other	26 (16.1%)
Paraprotein subtype	
IgG Kappa	48 (29.8%)
IgG Lambda	44 (27.3%)
IgA Kappa	22 (13.7%)
IgA Lambda	18 (11.2%)
Free Kappa	13 (8.1%)
Free Lambda	9 (5.6%)
Other/unknown	7 (4.3%)
ECOG performance status	
0–2	134 (83.2%)
3–4	12 (7.5%)
Not available	15 (9.3%)

IQR, interquartile range; ISS, International Staging System; VCD, bortezomib, cyclophosphamide, and dexamethasone; VRD, bortezomib, lenalidomide, and dexamethasone; VD/RD, bortezomib + dexamethasone/lenalidomide + dexamethasone; ECOG, Eastern Cooperative Oncology Group.

### Infection incidence and timing

**FIGURE 1:**
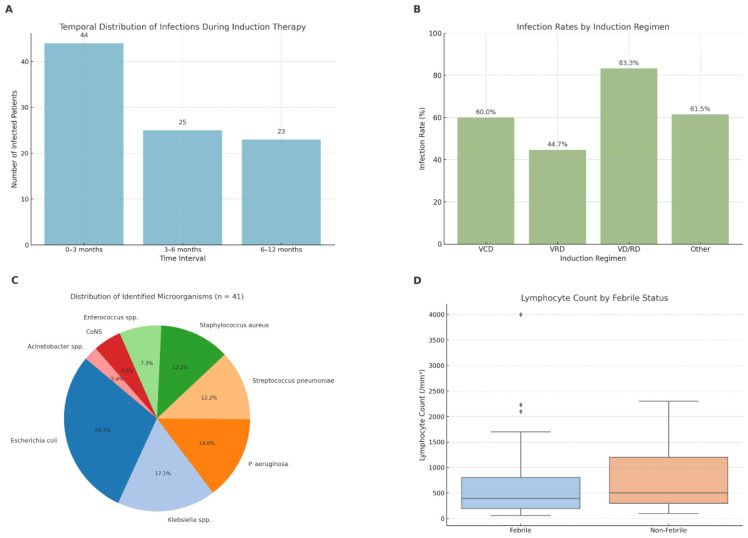
Infection incidence, etiology, and hematologic associations. **(A)** Temporal distribution of infections during the first year of induction therapy. Infections were most frequent in the first 3 months (*n* = 44), followed by 3–6 months (*n* = 25), and 6–12 months (*n* = 23). The difference in infection incidence across time intervals was statistically significant (*p* = 0.004). **(B)** Infection rates by induction regimen. The highest infection rate was observed in patients receiving VD/RD (83.3%), followed by other regimens (61.5%), VCD (60.0%), and VRD (44.7%). Although numerically different, the variation in infection rates among regimens did not reach statistical significance (*p* = 0.10). **(C)** Distribution of microorganisms isolated from 41 culture-positive infections. *Escherichia coli* was the most common pathogen (29.3%), followed by *Klebsiella* spp. (17.1%) and *P. aeruginosa* (14.6%), indicating a predominance of gram-negative bacteria. **(D)** Lymphocyte count by febrile status. Median lymphocyte count was significantly lower in febrile episodes compared to non-febrile ones (652/mmł vs. 805/mmł; *p* = 0.040), suggesting more profound immunosuppression in febrile patients.

During the first 12 months of induction therapy, 82 patients (50.9%) experienced at least one episode of infection. Infections were most frequent in the first 3 months (44 patients, 27.3%), followed by 3–6 months (25 patients, 15.5%) and 6–12 months (23 patients, 14.3%). Infection rates varied significantly across these time periods (*p* = 0.004) (Figure 1A), indicating increased vulnerability during the early phase of treatment. This temporal pattern further supports the concentration of infections in the early phase of treatment.

### Impact of induction regimens

Patients were categorized into four groups based on induction regimens: VCD (*n* = 85, 52.8%), VRD (*n* = 38, 23.6%), VD/RD (*n* = 12, 7.5%), and other protocols (*n* = 26, 16.1%). Infection rates were highest in the VD/RD group (83.3%), followed by “other” regimens (61.5%), VCD (60.0%), and VRD (44.7%). A limited number of patients received daratumumab-containing regimens (*n* = 5). Due to the small sample size, further subgroup analysis regarding infection risk in this population was not feasible. Although infection rates differed numerically, the overall difference among groups was not statistically significant (*p* = 0.10) (Figure 1B). The elevated infection rate in the VD/RD group may reflect patient selection for this regimen, which is often used in older or more comorbid individuals.

### Infection type and severity

Among the 82 infected patients, pneumonia was the most common infection (28 patients, 34.1%), followed by urinary tract infections (22 patients, 26.8%), infections without a clear focus (11 patients, 13.4%), and catheter-related infections (5 patients, 6.1%). Notably, 58.3% of infected patients (48 individuals) required hospitalization, indicating the clinical severity of these events (Table 2).

**TABLE 2:** Distribution and severity (Grade 3–4) of infections (*n* = 82).

Type of infection	*n* (%)	Grade 3–4 (%)
Pneumonia	28 (34.1%)	18 (64.3%)
UTI	22 (26.8%)	13 (59.1%)
No identifiable focus	11 (13.4%)	6 (54.5%)
Catheter-related infection	5 (6.1%)	5 (100%)
URTI	4 (4.9%)	1 (25%)
Soft tissue/local infection	4 (4.9%)	2 (50%)
Gastrointestinal infection	4 (4.9%)	2 (50%)
Other	4 (4.9%)	1 (25%)

UTI, urinary tract infection; URTI, upper respiratory tract infection. Grade 3–4 infections were defined according to Common Terminology Criteria for Adverse Events, version 5.0 (CTCAE v5.0).

### Microbiological findings

Among 41 culture-positive infections, *Escherichia coli* was the most frequently isolated pathogen (12 cases, 29.3%), followed by *Klebsiella* spp. (7 cases, 17.1%) and *Pseudomonas aeruginosa* (6 cases, 14.6%) (Figure 1C and Table 3). Gram-negative bacteria were predominant among all identified organisms. Among the *E. coli* isolates with available susceptibility data, 66.7% were susceptible to gentamicin, while 50% were resistant to TMP-SMX and none showed full susceptibility to levofloxacin.

**TABLE 3:** Distribution of identified microorganisms (*n* = 41).

Microorganism	*n* (%)
*Escherichia coli*	12 (29.3%)
*Klebsiella* spp.	7 (17.1%)
*Pseudomonas aeruginosa*	6 (14.6%)
*Streptococcus pneumoniae*	5 (12.2%)
*Staphylococcus aureus*	5 (12.2%)
*Enterococcus* spp.	3 (7.3%)
CoNS	2 (4.9%)
*Acinetobacter* spp.	1 (2.4%)

*E. coli*, *Escherichia coli*; CoNS, coagulase-negative *Staphylococcus*; *S. aureus*, *Staphylococcus aureus*; *S. pneumoniae*, *Streptococcus pneumoniae*.

### Hematologic parameters and febrile episodes

Neutrophil and lymphocyte counts at the time of infection were compared between febrile and non-febrile episodes. Neutrophil counts were lower in febrile episodes (median 3085/mmł vs. 3913/mmł), although the difference was not statistically significant (*p* = 0.084). In contrast, lymphocyte counts were significantly lower in febrile cases (median 652/mmł vs. 805/mmł; *p* = 0.040) (Figure 1D), suggesting a more profound immunosuppressive state.

### Multivariate analysis of infection risk

**FIGURE 2:**
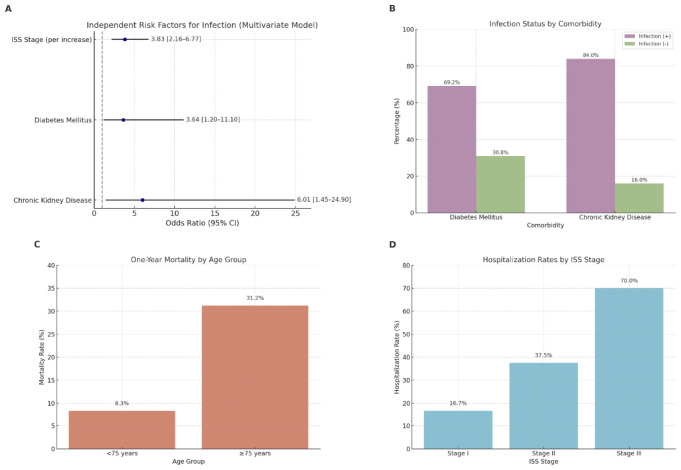
Infection and mortality risk by clinical parameters. **(A)** Multivariate logistic regression analysis showing independent risk factors for infection. Advanced ISS stage (OR: 3.83; 95% CI: 2.16–6.77; *p* < 0.001), diabetes mellitus (OR: 3.64; 95% CI: 1.20–11.10; *p* = 0.023), and chronic kidney disease (OR: 6.01; 95% CI: 1.45–24.90; *p* = 0.013) were significantly associated with infection risk. **(B)** Infection status by comorbidity. Patients with diabetes mellitus had a higher infection rate (69.2%) than those without (*p* = 0.019). Similarly, 84.0% of patients with chronic kidney disease developed infection compared to those without (*p* = 0.003). **(C)** One-year mortality by age group. Patients aged ≥ 75 years had significantly higher mortality (31.2%) compared to those <75 years (8.3%; *p* = 0.016), indicating age as a key predictor of early mortality. **(D)** Hospitalization rates by ISS stage. Hospitalization was significantly more common in patients with advanced disease: Stage I (16.7%), Stage II (37.5%), and Stage III (70.0%) (*p* < 0.001), emphasizing the role of disease burden in infection severity.

Multivariate logistic regression analysis identified advanced ISS stage, diabetes mellitus, and chronic kidney disease as independent predictors of infection. The odds of infection increased with each advancing ISS stage (OR: 3.83; 95% CI: 2.16–6.77; *p* < 0.001). Diabetes mellitus (OR: 3.64; 95% CI: 1.20–11.10; *p* = 0.023) and chronic kidney disease (OR: 6.01; 95% CI: 1.45–24.90; *p* = 0.013) were also significantly associated with infection risk. Age, sex, and hypertension were not independent predictors in the final model (Figure 2A and Table 4).

**TABLE 4:** Univariate and multivariate logistic regression analysis for infection risk.

Variable	Univariate OR	95% CI	*P*-value	Multivariate OR	95% CI	*P*-value
Age	1.02	0.99–1.06	0.248	1.03	0.99–1.09	0.162
Sex	0.65	0.32–1.32	0.236	1.01	0.42–2.41	0.987
Hypertension	1.50	0.74–3.03	0.258	0.65	0.25–1.70	0.376
Diabetes mellitus	2.13	0.93–4.88	0.073	3.64	1.20–11.09	0.023
CKD	6.33	1.75–22.88	0.005	6.02	1.45–24.88	0.013
ISS	3.51	2.13–5.80	0.000	3.83	2.16–6.77	0.000

OR, odds ratio; CI, confidence interval; CKD, chronic kidney disease; ISS, International Staging System.

### Infection risk by age, ISS stage, and comorbidities

Patients aged ≥ 75 years had a significantly higher infection rate compared to those under 75 (69.2% vs. 46.3%, *p* = 0.044). Infection rates also increased substantially with advancing ISS stage: 15.2% in stage I, 53.3% in stage II, and 72.3% in stage III (*p* < 0.0001). The presence of diabetes mellitus (*p* = 0.019) and chronic kidney disease (*p* = 0.003) were also significantly associated with increased infection risk (Figure 2B), whereas hypertension was not (*p* = 0.123).

### Mortality analysis

Seventeen patients (10.6%) died within the first year of treatment. Mortality was significantly higher in patients aged ≥ 75 years than in those younger than 75 (31.2% vs. 8.3%, *p* = 0.016) (Figure 2C). There was no significant difference in the number of comorbidities between patients who survived and those who died (median of 1 in both groups, *p* = 0.91). No individual comorbidity was significantly associated with mortality, though cardiovascular disease showed a trend toward significance (*p* = 0.085). Mortality rates by ISS stage were 9.1% (stage I), 3.3% (stage II), and 9.2% (stage III), but the difference was not statistically significant (*p* = 0.579), suggesting that factors such as age may have a stronger influence on early mortality.

### Hospitalization predictors

A separate analysis explored the relationship between hospitalization and clinical/laboratory variables. Higher ISS stage was significantly associated with hospitalization (*p* < 0.001) (Figure 2D), as was the presence of chronic kidney disease (*p* = 0.042). No significant associations were found for hypertension, diabetes mellitus, age, neutrophil count, or lymphocyte count.

## Discussion

Infections continue to represent a major clinical burden in patients with MM, particularly during the initial months of induction therapy. In our cohort, more than half of the patients (50.9%) experienced at least one infectious episode within the first year of treatment, with a significantly higher incidence during the first 3 months. This early vulnerability period is likely driven by a convergence of disease-related immune dysfunction, treatment-induced cytopenias, and underlying comorbid conditions (3).

Pneumonia was the most common type of infection, consistent with prior data highlighting respiratory tract susceptibility in MM. Notably, gram-negative pathogens, particularly *Escherichia coli* and *Klebsiella* species, were predominant in culture-positive cases. These findings support the hypothesis that gastrointestinal and urinary sources of infection are key contributors in this population (8). Although pathogen distribution was evaluated, we did not perform a stratified analysis based on the timing of infections due to the limited number of culture-positive episodes. Future studies with larger cohorts may help reveal potential temporal shifts in pathogen profiles, such as an early predominance of bacterial infections and later emergence of viral or fungal etiologies. The high rate of hospitalization (58.3%) among patients with infections further emphasizes the clinical severity and healthcare burden associated with infectious complications in MM. The absence of reported CMV infections in our cohort may partly reflect limited routine testing rather than a true absence of viral reactivation.

Although the number of antibiogram-tested isolates was limited, preliminary findings indicated potential resistance to commonly used agents such as TMP-SMX and levofloxacin. Among *E. coli* isolates, susceptibility to gentamicin was moderate (66.7%), while 50% were resistant to TMP-SMX, and none showed full susceptibility to levofloxacin. These data suggest that the empirical use of TMP-SMX or fluoroquinolones in MM patients should be approached with caution, consistent with recent findings on evolving resistance patterns in hematologic malignancies (14). Therefore, monitoring local antibiotic resistance patterns is essential in guiding empirical treatment decisions (8, 14). Larger-scale studies focusing on pathogen-specific resistance profiles in MM patients are warranted, especially given clinical data from Jena University Hospital, where over 65% of patients developed at least one infectious episode–primarily bacterial–during treatment with novel agents (15).

Age ≥ 75 years emerged as a significant predictor of both infection and 1-year mortality, underlining the importance of immunosenescence and frailty in clinical outcomes. Additionally, a higher ISS stage was strongly associated with both infection and hospitalization, reinforcing disease burden as a marker of immune compromise. In multivariate analysis, diabetes mellitus and chronic kidney disease were also found to independently increase the risk of infection, suggesting a need to incorporate these factors into individualized risk assessments. These findings are consistent with prior literature linking advanced age, disease stage, and organ dysfunction with heightened infectious risk in multiple myeloma patients (8, 16–18). In the multivariate model, although several variables were included based on clinical relevance and univariate screening, only ISS stage, diabetes mellitus, and chronic kidney disease remained statistically significant, supporting the robustness of these predictors. In our cohort, 33 patients were documented to have undergone autologous stem cell transplantation; however, the retrospective design and variability in the timing of transplantation in relation to infectious events limited our ability to assess its impact on infection incidence during the first year. Additionally, some transplantation procedures performed at external centers may not have been fully captured in our dataset.

Interestingly, although neutrophil counts did not significantly differ between febrile and non-febrile episodes, lymphopenia was significantly more pronounced in febrile infections. This observation suggests that lymphopenia may reflect a deeper state of immune suppression associated with clinically significant infections. While our retrospective design and limited sample size precluded establishing a precise threshold, the consistent trend underscores its potential as an early biomarker of infection risk. This is consistent with prior studies demonstrating that low absolute lymphocyte count (ALC) is associated with increased infection risk and inferior outcomes in multiple myeloma patients (19, 20). Future prospective studies are warranted to validate lymphocyte count as a stratification tool for infection surveillance and preemptive interventions in MM patients.

When comparing induction regimens, no statistically significant differences in infection or mortality rates were observed. However, the highest infection rate was seen in the VD/RD group (83.3%), which may reflect the preferential use of this regimen in older, more frail patients. Similarly, mortality rates were numerically higher in this group, though not statistically significant. These trends underscore the importance of considering patient selection and baseline characteristics when interpreting treatment-related outcomes. This observation is consistent with previous studies showing that frailty is a key determinant of infectious complications and early mortality in transplant-ineligible multiple myeloma patients (21, 22).

Although daratumumab-based regimens are increasingly used in multiple myeloma, only a small number of patients in our cohort received these treatments (*n* = 5). Therefore, we were unable to draw definitive conclusions regarding their impact on infection risk. Future studies with larger patient populations are needed to further explore this association.

Hospitalization was more frequently required in patients with advanced ISS stage and in those with chronic kidney disease. Other parameters such as age, neutrophil and lymphocyte counts, and additional comorbidities were not independently associated with the need for inpatient care. These findings suggest that disease burden and renal impairment more accurately reflect clinical vulnerability than age or isolated laboratory markers in MM patients. This aligns with previous reports indicating that advanced disease stage and renal dysfunction predict hospitalization and infectious complications, while age and isolated cytopenias are weaker predictors (23, 24).

While antibiotic prophylaxis has been proposed as a strategy to mitigate infection risk in MM, its routine use remains controversial. The TEAMM trial demonstrated that levofloxacin prophylaxis significantly reduced febrile episodes and early mortality within the first 12 weeks of treatment in newly diagnosed MM patients (5). However, concerns regarding antibiotic resistance, *C. difficile* infection, and microbiome disruption have limited widespread adoption. In our cohort, none of the patients received prophylactic antibiotics; yet, over half experienced infections, predominantly within the first 3 months. These findings underscore the importance of risk-adapted prevention strategies guided by patient-specific factors such as ISS stage, age, and comorbidities, rather than routine blanket prophylaxis (25–27).

Given the predominance of gram-negative pathogens in our cohort, targeted prophylaxis with levofloxacin may be considered as a hypothesis-generating approach for high-risk patients. However, this suggestion should be interpreted with caution, as our data do not directly support it. Such strategies remain speculative and require validation in prospective studies (5, 9, 28).

## Conclusion

This study highlights the substantial burden of infections during the first year of multiple myeloma treatment, particularly within the early phases of induction therapy. Readily available clinical parameters–such as advanced ISS stage, diabetes mellitus, and chronic kidney disease–were identified as independent predictors of infection and hospitalization. These factors may serve as practical tools for early risk stratification and may help inform the development of personalized preventive strategies.

Our findings emphasize the importance of individualized, risk-adapted infection prevention strategies during the early treatment phase. By incorporating clinical risk factors into decision-making, healthcare providers may improve surveillance, antimicrobial stewardship, and supportive care.

However, given the single-center design and the limited number of culture-positive infections, our findings should be interpreted with caution and validated through larger, prospective multicenter studies.

## Limitations

This study has several limitations that should be acknowledged. First, its single-center retrospective design may limit generalizability and introduce potential selection bias. Second, missing data–particularly regarding cytogenetics and LDH–prevented the calculation of Revised ISS scores, potentially affecting the precision of prognostic stratification. Third, not all infectious episodes were microbiologically confirmed; thus, reliance on clinical judgment may have led to potential misclassification of infection events. Furthermore, the observational design of the study may have introduced potential confounding by indication, as treatment decisions were influenced by baseline patient characteristics, which may have affected the observed associations between clinical variables and infection outcomes. Due to the retrospective structure of the dataset and inconsistencies in the documentation of exact event timing, median time to first infection could not be reliably calculated.

Additionally, treatment protocols and supportive care practices may have evolved over the extended study period, introducing potential heterogeneity. The cause of death was not systematically documented, limiting our ability to determine infection-related versus disease-related mortality. In addition, data on baseline immunoglobulin levels were not consistently available, precluding assessment of hypogammaglobulinemia as a potential risk factor for infection. Information on immunoglobulin replacement therapy (IVIG) was also not systematically recorded and could not be evaluated in the analysis.

Nevertheless, our findings provide meaningful real-world insights into infection patterns and associated risk factors in newly diagnosed MM patients, and may help inform future prospective studies aimed at improving risk stratification and preventive strategies.

## Data Availability

The raw data supporting the conclusions of this article will be made available by the authors, without undue reservation.

## References

[1] Ying L YinHui T Yunliang Z Sun H. Lenalidomide and the risk of serious infection in patients with multiple myeloma: a systematic review and meta-analysis. *Oncotarget.* (2017) 8:46593–600. 10.18632/oncotarget.16235 28423741 PMC5542295

[2] Blimark C Holmberg E Mellqvist U-H Landgren O Björkholm M Hultcrantz M et al.. Multiple myeloma and infections: a population-based study on 9253 multiple myeloma patients. *Haematologica.* (2015) 100:107–13. 10.3324/haematol.2014.107714 25344526 PMC4281323

[3] Blimark CH Carlson K Day C Einarsdottir S Juliusson G Karma M et al.. Risk of infections in multiple myeloma. A populationbased study on 8,672 multiple myeloma patients diagnosed 2008-2021 from the Swedish Myeloma Registry. *Haematologica.* (2024) 110:163–72. 10.3324/haematol.2024.285645 39021214 PMC11694133

[4] Sørrig R Klausen TW Salomo M Vangsted A Gimsing P. Risk factors for infections in newly diagnosed multiple myeloma patients: a Danish retrospective nationwide cohort study. *Eur J Haematol.* (2019) 102:182–90. 10.1111/ejh.13190 30485563

[5] Drayson MT Bowcock S Planche T Iqbal G Pratt G Yong K et al.. Levofloxacin prophylaxis in patients with newly diagnosed myeloma (TEAMM): a multicentre, double-blind, placebo-controlled, randomised, phase 3 trial. *Lancet Oncol.* (2019) 20:1760–72. 10.1016/S1470-2045(19)30506-6 31668592 PMC6891230

[6] Teh BW Harrison SJ Worth LJ Spelman T Thursky KA Slavin MA. Risks, severity and timing of infections in patients with multiple myeloma: a longitudinal cohort study in the era of immunomodulatory drug therapy. *Br J Haematol.* (2015) 171:100–8. 10.1111/bjh.13532 26105211

[7] Russell BM Avigan DE. Immune dysregulation in multiple myeloma: the current and future role of cell-based immunotherapy. *Int J Hematol.* (2023) 117:652–9. 10.1007/s12185-023-03579-x 36964840 PMC10039687

[8] Balmaceda N Aziz M Chandrasekar VT McClune B Kambhampati S Shune L et al.. Infection risks in multiple myeloma: a systematic review and meta-analysis of randomized trials from 2015 to 2019. *BMC Cancer.* (2021) 21:730. 10.1186/s12885-021-08451-x 34172037 PMC8233183

[9] Raje NS Anaissie E Kumar SK Lonial S Martin T Gertz MA et al.. Consensus guidelines and recommendations for infection prevention in multiple myeloma: a report from the International Myeloma Working Group. *Lancet Haematol.* (2022) 9:e143–61. 10.1016/S2352-3026(21)00283-0 35114152

[10] Ticona K Tun A Guevara E. Risks of upper respiratory tract infection and pneumonia in patients with multiple myeloma receiving Daratumumab: a systematic review and meta-analysis of randomized controlled trials. *Am Soc Clin Oncol.* (2018) 36:e20005. 10.1200/JCO.2018.36.15_suppl.e20005

[11] Tobar P Gonzalez Mosquera LF Cardenas Maldonado DD Moscoso B Podrumar AI Cuenca JA. Clinical and financial implications of central venous catheters bloodstream infections in patients with multiple myeloma in the United States. *J Clin Oncol.* (2021) 39:e20033. 10.1200/JCO.2021.39.15_suppl.e20033

[12] Teh BW Teng JC Urbancic K Grigg A Harrison SJ Worth LJ et al.. Invasive fungal infections in patients with multiple myeloma: a multi-center study in the era of novel myeloma therapies. *Haematologica.* (2015) 100:e28–31. 10.3324/haematol.2014.114025 25304609 PMC4281332

[13] Taplitz RA Kennedy EB Bow EJ Crews J Gleason C Hawley DK et al.. Outpatient management of fever and neutropenia in adults treated for malignancy: American Society of Clinical Oncology and Infectious Diseases Society of America clinical practice guideline update. *J Clin Oncol.* (2018) 36:1443–53. 10.1200/JCO.2017.77.6211 29461916

[14] Guevara-Ramírez P Cadena-Ullauri S Paz-Cruz E Ruiz-Pozo VA Tamayo-Trujillo R Cabrera-Andrade A et al.. Gut microbiota disruption in hematologic cancer therapy: molecular insights and implications for treatment efficacy. *Intern J Mol Sci.* (2024) 25:10255. 10.3390/ijms251910255 39408584 PMC11476909

[15] Brioli A Nägler TM Yomade O Rüthrich MM Scholl S Frietsch JJ et al.. Sex-disaggregated analysis of biology, treatment tolerability, and outcome of multiple myeloma in a German cohort. *Oncol Res Treatment.* (2022) 45:494–503. 10.1159/000525493 35705004

[16] Moore KLF Turesson I Genell A Klausen TW Knut-Bojanowska D Redder L et al.. Improved survival in myeloma patients-a nationwide registry study of 4,647 patients ≥75 years treated in Denmark and Sweden. *Haematologica.* (2022) 108:1640–51. 10.3324/haematol.2021.280424 36300775 PMC10230423

[17] Lim C Sinha P Harrison SJ Quach H Slavin MA Teh BW. Epidemiology and risks of infections in patients with multiple myeloma managed with new generation therapies. *Clin Lymphoma Myeloma Leukemia.* (2021) 21:444–50.e3. 10.1016/j.clml.2021.02.002 33722538

[18] Palumbo A Bringhen S Ludwig H Dimopoulos MA Bladé J Mateos MV et al.. Personalized therapy in multiple myeloma according to patient age and vulnerability: a report of the European Myeloma Network (EMN). *Blood.* (2011) 118:4519–29. 10.1182/blood-2011-06-358812 21841166

[19] Ferri GM Yildirim C Do NV Brophy M Park JS Munshi NC et al.. Lymphopenia predicts poor outcomes in newly diagnosed multiple myeloma. *Blood Adv.* (2025) 9:78–88. 10.1182/bloodadvances.2024014125 39471425 PMC11742561

[20] Allegra A Tonacci A Musolino C Pioggia G Gangemi S. Secondary immunodeficiency in hematological malignancies: focus on multiple myeloma and chronic lymphocytic leukemia. *Front Immunol.* (2021) 12:738915. 10.3389/fimmu.2021.738915 34759921 PMC8573331

[21] Spataro F Armentaro G Di Gioia G Meloni P Rossi I Williams M et al.. Impact of frailty on infection risk in non-transplant eligible multiple myeloma patients: a systematic review and meta-analysis. *Leukemia.* (2026): [Epub ahead of print]. 10.1038/s41375-026-02880-y 41703031 PMC13149299

[22] Offidani M Corvatta L Bringhen S Gentili S Gay F Maracci L et al.. *Infection Complications in 476 Patients with Newly Diagnosed Multiple Myeloma Treated with Lenalidomide or Bortezomib Combinations.* Washington, DC: American Society of Hematology (2015).

[23] Valković T Gačić V Ivandić J Petrov B Dobrila-Dintinjana R Dadić-Hero E et al.. Infections in hospitalised patients with multiple myeloma: main characteristics and risk factors. *Turk J Haematol.* (2015) 32:234–42. 10.4274/tjh.2013.0173 26376590 PMC4563199

[24] Wei W Shi H Chen H Chen X Peng R Yu W et al.. Clinicopathologic predictors of renal response and survival in newly diagnosed multiple myeloma with renal injury: a retrospective study. *Clin Exp Med.* (2025) 25:48. 10.1007/s10238-025-01571-9 39904814 PMC11794406

[25] Sedhom D Elsaid M Sedhom R. Clostridium difficile infection in patients with hematologic malignancy and neutropenic fever: a clinical overview: 149. *Am Coll Gastroenterol.* (2018) 113:S83–4. 10.14309/00000434-201810001-00149

[26] Freifeld AG Bow EJ Sepkowitz KA Boeckh MJ Ito JI Mullen CA et al.. Clinical practice guideline for the use of antimicrobial agents in neutropenic patients with cancer: 2010 update by the Infectious Diseases Society of America. *Clin Infect Dis.* (2011) 52:e56–93. 10.1093/cid/cir073 21258094

[27] Ziegler M Han JH Landsburg D Pegues D Reesey E Gilmar C et al.. Impact of levofloxacin for the prophylaxis of bloodstream infection on the gut microbiome in patients with hematologic malignancy. *Open Forum Infect Dis.* (2019) 6:ofz252. 10.1093/ofid/ofz252 31281857 PMC6602896

[28] Satlin MJ Vardhana S Soave R Shore TB Mark TM Jacobs SE et al.. Impact of prophylactic levofloxacin on rates of bloodstream infection and fever in neutropenic patients with multiple myeloma undergoing autologous hematopoietic stem cell transplantation. *Biol Blood Marrow Trans.* (2015) 21:1808–14. 10.1016/j.bbmt.2015.06.017 26150022 PMC4568152

